# Co-Infection Dynamics of Baculovirus Penaei (BP–PvSNPV) in *Penaeus vannamei* Across Latin America

**DOI:** 10.3390/v17030374

**Published:** 2025-03-05

**Authors:** Pablo Intriago, Bolivar Montiel, Mauricio Valarezo, Nicole Cercado, Alejandra Montenegro, María Mercedes Vásquez, Melany del Barco, Yamilis Cataño

**Affiliations:** 1 South Florida Farming Corp, 13811 Old Sheridan St, Southwest Ranches, FL 33330, USA; 2 South Florida Farming Lab, Av. Miguel Yunez, Km 14.5 Via A Samborondón, Almax 3 Etapa 1-Lote 3 Bodega 2, Samborondón, Guayas, Ecuador; bolivarmontielr@gmail.com (B.M.); mauriciovalarezogilbert@gmail.com (M.V.); nicolecercado06@gmail.com (N.C.); alejandramontenegro1020@outlook.com (A.M.); maimev17@hotmail.com (M.M.V.); delbarcomelany@gmail.com (M.d.B.); 3 Océanos S.A., Centro de Producción Laboratorio, Coveñas, Sucre, Colombia; silimaya@yahoo.es

**Keywords:** *Penaeus vannamei*—Pv, Baculovirus penaei-single enveloped nucleopolyhedron virus (BP—PvSNPV), Hepatopancreas—HP, Wenzhou shrimp virus 8—WzSV8, Hepanhamaparvovirus—DHPV, Postlarvae—PL, Viral inclusion—VIN

## Abstract

Baculovirus penaei (BP) is an enteric virus infecting the hepatopancreas and anterior midgut of shrimp, particularly affecting early developmental stages and contributing to hatchery losses. While BP’s role in co-infections is increasingly recognized, its impact on later life stages remains unclear. Despite advancements in molecular diagnostics, its high genetic diversity complicates reliable detection, often leading to discrepancies between PCR results and histological observations of occlusion bodies. This study evaluated seven primer pairs for BP detection in *Penaeus vannamei*. Among histologically confirmed cases, only 6% tested positive with the BPA/BPF primer and 3% with BPA/BPB, while the remaining primers failed to amplify BP, highlighting significant diagnostic limitations. Histopathology revealed bacterial co-infections alongside BP, with advanced cases showing intranuclear occlusion bodies, hepatopancreatic necrosis, and epithelial detachment. These findings underscore the urgent need for improved molecular diagnostics to accurately assess BP prevalence, its role in co-infections, and its overall impact on shrimp health in Latin America. Further research is essential to refine detection methods and determine BP’s pathogenic significance beyond early developmental stages.

## 1. Introduction

*Baculovirus penaei* (BP) is a double-stranded DNA virus classified among the nuclear polyhedrosis viruses (NPVs). These viruses are divided into two groups based on the morphology of their occlusion bodies (OBs): MBV-type viruses, which are characterized by rounded intranuclear OBs and are more closely related to nudiviruses than to baculoviruses [1,2], and BP-type viruses, which feature tetrahedral OBs. BP-type viruses, notable for their distinct OB morphology, have been documented in 15 penaeid shrimp species and are widely distributed throughout the Americas [3,4].

BP, also known as the singly enveloped nucleopolyhedrosis virus of *Penaeus vannamei* (PvSNPV) [5], has virions measuring approximately 75 × 300 nm in size [6]. It is strictly enteric, targeting the mucosal epithelial cells of the hepatopancreas tubules and the anterior midgut [3,7,8,9,10,11,12]. The geographic range of BP has historically been confined to the Americas and Hawaii, where it is enzootic in wild penaeid populations. However, in 2022, BP was reported for the first time outside the Americas, with an outbreak identified at a shrimp farm in northern Taiwan [13].

In *P. vannamei* shrimp carrying the BP virus, infection can persist in the environment due to host excretion of waste; this is the case with adult females that defecate during spawning, facilitating the transmission of the virus to subsequent generations [11]. It has been observed that the larval stages of *P. vannamei* (zoea, mysis, and post-larva) are more likely to be infected, with high mortality in incubation systems [14,15,16,17,18,19,20]. While high mortality is uncommon in juvenile or adult stages, BP infections can lead to poor growth performance and reduced survival rates in nursery and grow-out ponds at shrimp farms [12]. All BP-exposed groups of early postlarvae (PL 9 or younger) became heavily infected within 2–5 days of initial exposure to the virus. Some of these groups experienced high mortalities compared to the noninfected controls. Postlarvae that survived the infection exhibited highly variable and significantly reduced growth, as determined by dry weight, compared to controls [18].

The coinfection of BP with other pathogens is not uncommon. Ramirez et al. [21] reported the coinfection of two enteric pathogens, BP and *Hepatobacter penaei* (NHP), and two systemic pathogens (infectious hypodermal and haematopoietic necrosis virus, IHHNV, and white spot syndrome virus, WSSV) in wild shrimp *P. vannamei* and *P. stylirostris* from seven tidal channels of mangroves in Tumbes, Peru. Varela-Mejías [22] reported the presence of BP in postlarvae imported to Central America. The role of BP as an important risk factor for bacterial infections has also been previously highlighted by [23].

BP was classified as a C2 pathogen, highlighting its significance and the potential need for exclusion from aquaculture systems [24]. According to this classification, C1 pathogens are those that should be prevented due to their potential to cause catastrophic losses in one or more American penaeid species, C2 pathogens are considered serious and potentially avoidable, while C3 pathogens have minimal impact [24].

Nonetheless, due to the lack of substantial or relevant reports on its pathogenic impact, BP was removed from the WOAH list of notifiable aquatic animal diseases in 2009 [25]. To detect the genome of BP, several molecular diagnostic methods have been developed, including in situ hybridization (ISH), which links lesions to BP infection, and polymerase chain reaction (PCR) [26,27,28,29]. Despite these advancements, Cheng et al. [13] reported that the primers described by Wang et al. [26] failed to amplify PCR products, suggesting high genetic diversity among BP strains, particularly in the polyhedrin genes. This genetic variability may contribute to inconsistencies between molecular diagnostics and the distinct polyhedral occlusion bodies observed in histological analyses.

Although BP infection has been documented in the Americas for nearly four decades [4,29,30], only one partial BP nucleotide sequence is available in GenBank (DQ496179.1). This sequence, originating from Hawaii, USA, represents the Pacific strain, which differs from the Hawaiian strain found in native *Penaeus marginatus*. According to [4], this virus was isolated from a population of *P. vannamei* originally imported from Ecuador and subsequently cultured in Hawaii.

This study seeks to address these challenges by correlating molecular findings with histological evidence of *Baculovirus penaei* (BP) infection. Furthermore, it aims to validate the findings by [13], which highlighted the lack of specificity in previously published primers, and to evaluate the applicability of current molecular tools across diverse geographical contexts.

To achieve these objectives, several samples of *P. vannamei* post-larvae (PL) and broodstock from multiple Latin American countries were submitted to our laboratory for comprehensive histopathological and PCR analysis. A total of twenty primers targeting various pathogens, including viruses and bacteria, were tested. Although no mortality was reported, these samples were submitted for diagnostic investigation due to suspected infections. The Following Pathogens Were Screened: Wenzhou shrimp virus 8 (WzSV8); hepanhamaparvovirus (DHPV); Macrobrachium bidnavirus (MrBdv); rickettsia-like bacteria (RLB); necrotizing hepatopancreatitis bacteria (NHP-B); Spiroplasma; non-EHP microsporidia; infectious hypodermal and hematopoietic necrosis virus (IHHNV); Enterocytozoon hepatopenaei (EHP); Vibrio spp.; Acute hepatopancreatic necrosis (AHPND); decapod iridescent virus 1 (DIV1); white spot syndrome virus (WSSV); Penaeus vannamei noda virus (PvNV); covert mortality nodavirus (CMNV); infectious myonecrosis virus (IMNV); yellow head virus (YHV); Taura syndrome virus (TSV); and Machrobrachium Nodavirus (MrNV). This provided a unique opportunity to assess the diagnostic reliability of molecular and histological methods under real-world field conditions.

## 2. Materials and Methods

Diagnosis of BP infections is achieved by demonstrating single or multiple polyhedral/tetrahedral occlusion bodies in the nuclei of epithelial cells in squash preparations of the hepatopancreas, midgut, or fecal samples. These preparations are examined using phase-contrast or bright-field microscopy [12,28,31]. Routine histological stains, such as hematoxylin and eosin (H&E), can provide a definitive diagnosis of BP infection. Typically, BP-infected hepatopancreatic (or occasionally midgut) cells exhibit markedly hypertrophied nuclei with single or, more often, multiple eosinophilic occlusion bodies, along with chromatin diminution and margination [12]. Additionally, the polymerase chain reaction (PCR) method, modified and described by [26], is employed for diagnosis.

### 2.1. Sample Collection

Samples for histopathology and PCR were received from different farms and maturation units in Latin America (LA). The shrimp used for PCR and histology were different individuals from the same populations, as pathogen loads in aquaculture are unevenly distributed across tissues. This variability can affect PCR detection, histopathological findings, and pathogen isolation, depending on the sampled tissue [32,33,34]. Factors such as infection severity and tissue tropism further influence diagnostic accuracy. Therefore, a careful selection of tissue types and sampling methods is essential to optimize PCR sensitivity and minimize false negatives. Despite using different shrimp for each analysis, all samples were derived from the same pond and exhibited similar symptoms.

It is important to clarify that our laboratory operates as a commercial unit providing services to farmers. The samples we process originate from commercial farms, hatcheries, and maturation facilities. We rely on the cooperation of our clients to supply adequate samples and share relevant information about the conditions on their farms. However, due to concerns about reputation, critical information, including details of disease outbreaks, is often withheld. Despite this, our laboratory consistently performs comprehensive analyses, PCR, histology, and, when applicable, microbiology, without specific requests to gain a better understanding of the pathology of the samples received. Additionally, to maintain client confidentiality, the countries or specific locations of the samples will not be disclosed. However, clients from the World Organization for Animal Health (WOAH) member countries are reminded of their responsibility to inform their respective authorities of any positive test results for shrimp pathogens listed by WOAH or any unusual mortality events. It is then the responsibility of these authorities to report such cases to the WOAH.

### 2.2. PCR Methods Used

The methods for DNA and RNA extraction, along with the sampling procedures, are detailed in [35,36] and referenced in Table 1, Table 2 and Table 3.

DNA was extracted from whole larvae, tissue, or organs fixed in 90% alcohol, following the manufacturer’s protocol (Omega, Bio-Tek E.Z.N.A. tissue DNA kit; Omega, Bio-Tek Inc. Norcross, GA, USA). In brief, each sample was minced with sterilized scissors and then ground using a microcentrifuge pestle. Approximately 200 mg of tissue was then transferred to a clean 1.5 mL Eppendorf tube. To this end, 500 μL of tissue lysis buffer (TL) and 25 μL of Omega Biotek (OB) protease solution were added, and the mixture was vortexed and then incubated in a thermoblock at 55 °C for approximately 3 h, with vortexing every 30 min. RNA was removed by adding 4 μL of RNase A (100 mg/mL), and after mixing, the sample was kept at room temperature for 2 min. The sample was then centrifuged at 13,500 RPM for 5 min, and the supernatant was carefully transferred to a new 1.5 mL Eppendorf tube. Then, 220 μL of BL buffer was added, and the mixture was vortexed and incubated at 70 °C for 10 min. Next, 220 μL of 100% ethanol was added, the mixture was vortexed, and the contents were passed through a HiBind^®^ DNA Mini Column into a 2 mL collection tube. The columns were then centrifuged at 13,500 RPM for 1 min, after which the filtrate was discarded. Subsequently, 500 μL of HBC buffer (diluted with 100% isopropanol) was added to the column, and the sample was spun at 13,500 RPM for 30 s. The filtrate was discarded, the column was washed twice with 700 μL of DNA wash buffer diluted with 100% ethanol, and the sample was centrifuged at 13,500 RPM for 30 s. The filtrate was discarded. This step was repeated. The column was then centrifuged at 13,500 RPM for 2 min to dry it. The dried column was placed in a new nuclease-free 1.5 mL Eppendorf tube, and 100 μL of elution buffer, which was heated to 70 °C, was added to the column. The sample was allowed to sit for 2 min before being centrifuged at 13,500 RPM for 1 min. This elution step was repeated. The eluted DNA was then stored at −20 °C until needed.

RNA was extracted from whole larvae, tissue, or organs fixed in 90% alcohol, following the manufacturer’s protocol (Omega, Bio-Tek E.Z.N.A. Total RNA Kit). In brief, each sample was minced with sterilized scissors and then ground using a microcentrifuge pestle. Approximately 200 mg of tissue was then transferred to a clean 1.5 mL Eppendorf tube. Then, 700 μL of TRK Lysis Buffer was added, and the tube was left at room temperature for approximately 3 h, with vortexing every 30 min. The sample was then centrifuged at 13,500 RPM for 5 min, and the supernatant was carefully transferred to a new 1.5 mL Eppendorf tube, to which 420 μL of 70% ethanol was added. After vortexing to mix thoroughly, the contents were passed through a HiBind^®^ RNA Mini Column into a 2 mL collection tube. The columns were then centrifuged at 13,500 RPM for 1 min, after which the filtrate was discarded. Subsequently, 500 μL of RNA Wash Buffer I was added to the column, and the sample was spun at 13,500 RPM for 30 s. The filtrate was discarded, and the column was washed twice with 500 μL of RNA Wash Buffer II and diluted with 100% ethanol. The column was then centrifuged at 13,500 RPM for 1 min to dry it. The filtrate was discarded. This step was repeated. The column was then centrifuged at 13,500 RPM for 2 min to dry it. The dried column was placed in a new nuclease-free 1.5 mL Eppendorf tube, and 70 μL of nuclease-free water was added to the column. The sample was centrifuged at 13,500 RPM for 2 min. This elution step was repeated. The eluted RNA was then stored at −70 °C until needed. The samples used for extraction were as follows:

A total of 20 pathogens were screened using PCR to identify or rule out potential pathogens that could correlate with our study or the histological analysis of samples from the same population. Detailed information on the primers used and corresponding amplicon sizes is provided in Table 2. PCR products were separated by 2% agarose gel electrophoresis, stained with 1 µL SYBR Safe Gel Stain, and visualized using a dual LED blue/white light transilluminator.

The combinations of Wang et al. [26] primers amplify segments from BP template DNA of the following: BPA/BPF—196 bp; BPA/BPB—560 bp; BPA/BPG—933 bp; BPD/BPB—207 bp; BPD/BPG—580 bp; and BPE/BPG—221 bp. We also tested the alternative method used by the WOAH Reference Laboratory at the University of Arizona [28]. This method used one forward and reverse primer pair that produces a 644 bp amplicon [28].

Table 3 outlines the sampling strategy for each individual PCR analysis.

### 2.3. Histopathology

For histological analysis, samples were prepared following the procedures outlined by [59]. Briefly, they were fixed in Davidson’s AFA for at least 24 h before processing for routine histological analysis. Next, 2–4 paraffin blocks were prepared and tissue sections of 5 µm thickness were stained with H&E-phloxine, as indicated by [60]. In addition, a methyl green pyronin modified stain was employed to distinguish DNA and RNA (Poly Scientific R&D Corp, Bay Shore, NY, USA).

## 3. Results

### 3.1. PCR Results

In this study, six primer pair combinations targeting putative *Baculovirus penaei* (BP) polyhedrin cDNA sequences, developed by Wang et al. [26], along with the primer set recommended by WOAH [28], were evaluated. Among the tested pairs—BPD/BPB, BPD/BPG, and BPE/BPG—none successfully amplified PCR products, underscoring the challenges in BP detection using these primers. Of the histologically positive samples, only 6% were PCR-positive with the BPA/BPF primer pair and 3% with the BPA/BPB primer pair, further highlighting the limitations of current molecular diagnostics for BP.

The detection of *Baculovirus penaei* (BP) via PCR is influenced by the quantity of BP DNA relative to the total DNA extracted from infected *P. vannamei* post-larvae. Wang et al. [26] reported that amplification failed when 5 ng of template DNA was used but was successful with a minimum of 10 ng, highlighting the sensitivity threshold of the assay. In our study, no false positives or nonspecific amplification products were observed even when using up to 200 ng of total DNA, suggesting the reliability of the PCR conditions employed.

Of the 20 pathogens listed in Table 1, only those in Table 4 were detected in the analyzed samples. Among the 33 samples tested, four DNA viruses, one RNA virus, and *Vibrio* spp. were identified. *WzSV8* was the most prevalent, present in 73% of all samples, followed by *DHPV* and *WSSV*, each detected in 30% of cases. *Vibrio* spp. was found in 18% of the samples, while *IHHNV* and *BP* had the lowest prevalence, at just 6%.

### 3.2. Histopathology Results

**Figure 1:**
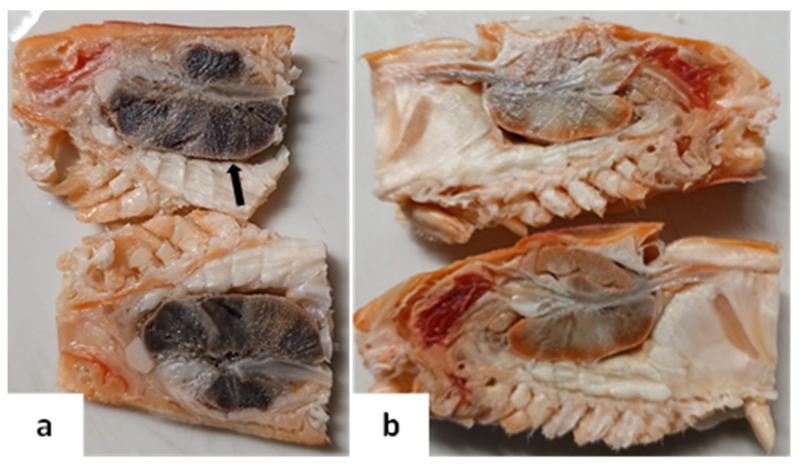
Sagittal view of the shrimp cephalothorax fixed in Davidson’s solution showing internal organs. (**a**) BP co-infected animal with dark appearance in the hepatopancreas (arrow), caused by a severe melanized reaction, necrosis, and epithelial detachment. (**b**) Normal animal.

Similarly to what was described by Cheng et al. [13], BP-infected animals did not exhibit any macroscopic lesions—meaning no visible changes detectable to the naked eye that are uniquely indicative of BP infection. This observation specifically pertains to BP and does not imply the absence of gross lesions in other diseases or conditions affecting shrimp. Histopathological examination appeared normal in cases of minor infection. Nonetheless, advanced co-infections revealed a dark coloration in the hepatopancreas during the dissection process (Figure 1). Initially, this discoloration was suspected to result from improper fixation. However, histological analysis revealed that the darkened appearance was attributable to necrotic damage in the hepatopancreas.

Table 5 is crucial as it corroborates the molecular findings and provides insight into host–pathogen interactions in BP infections. The presence of lymphoid organ spheroids, muscle necrosis, and melanized reactions in the exoskeleton suggests that additional pathogens contribute to these abnormalities. These histopathological observations support the conclusion that bacterial infections play a significant role in facilitating the progression of BP to advanced infection stages.

**Figure 2:**
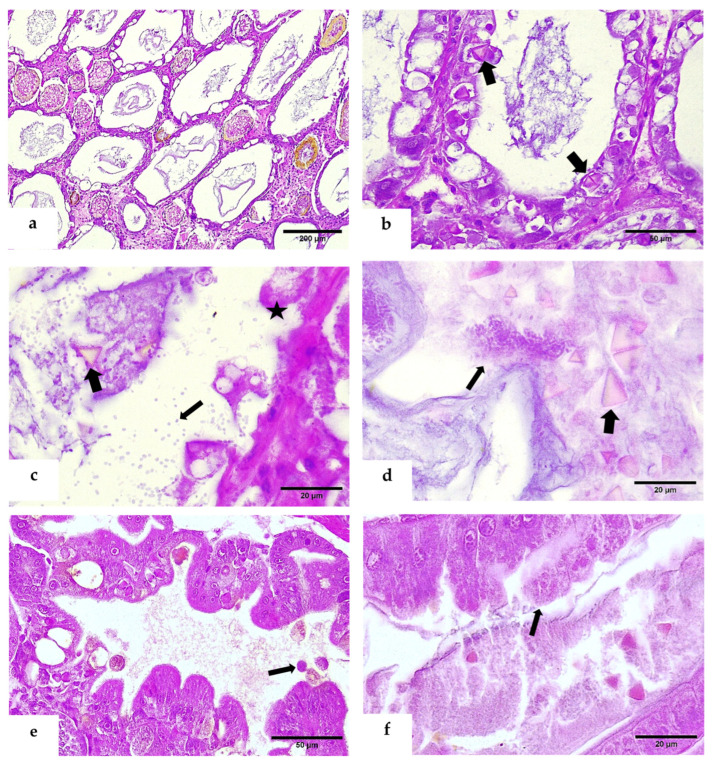
H&E-phloxine stained sections. (**a**) General view of the BP co-infected hepatopancreas. (**b**) Intranuclear tetrahedral eosinophilic occlusion bodies in hepatopancreatic epithelial cells (thick arrows). (**c**) Free OBs in the tubular lumen (thick arrow), epithelial detachment (star), and a significant presence of bacteria (thin arrow) in the hepatopancreas. (**d**) Intestinal lumen reveals BP tetrahedral occlusion bodies (thick arrow) and bacterial colonies (thin arrow). (**e**,**f**) Loss of epithelial cells (thin arrows) in the hepatopancreas and intestine of a post-larva infected by *Baculovirus penaei*.

Further examination revealed co-infections of bacterial pathogens and BP, ranging from mild to advanced stages. In advanced infections, free tetrahedral occlusion bodies were observed in the stomach lumen, indicating clear evidence of horizontal transmission. In the hepatopancreas, there was a moderate dilation of the tubules lacking vacuoles with foci of hemocyte infiltration in the interstitium, melanized/necrotic reaction, and detachment of epithelial cells, which contained one or more intranuclear tetrahedral occlusion bodies that were prominent within or budding out of the hypertrophic nucleus (Figure 2a–c). Bacterial colonies and BP occlusions were identified in the intestinal lumen (Figure 2d), while infected post-larvae (PL) exhibited important epithelial loss in the hepatopancreas and intestine (Figure 2e,f).

**Figure 3:**
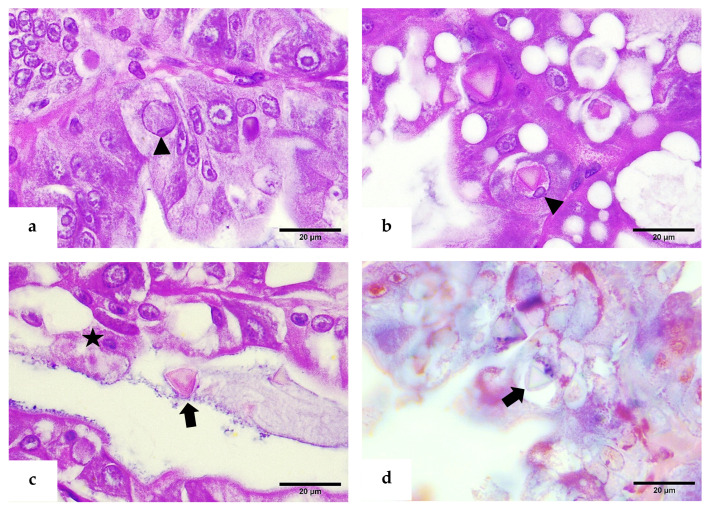
H&E-phloxine stained sections. (**a**,**b**) Hypertrophied nuclei with reduced chromatin, note the displacement of the nucleolus (arrowhead). (**c**) Free tetrahedral occlusion body in lumen of the hepatopancreas (thick arrow), and detachment of tissue (star). (**d**) Methyl green pyronin-stained section showing the tetrahedral body of PvSNPV with bright green coloration (thick arrow).

In addition to being hypertrophied, the infected nuclei were hypochromatic, with marginated nucleoli (Figure 3a,b). The occlusion bodies were approximately 7 μm in diameter and were commonly observed in the tubular lumen of the hepatopancreas (Figure 3c). Tetrahedral OBs were stained bright green with methyl green staining (Figure 3d). No similar lesions, such as intranuclear occlusion bodies, swollen nuclei, or fragmented nucleoli, were observed in the distal regions of hepatopancreatic tubules or midgut epithelial cells.

### 3.3. Linking PCR with Histopathology Results

Typical of shrimp from the region, the dominant pathology involves hepatopancreatic lesions associated with bacterial infections, with WzSV8 frequently acting as a co-infectious agent [35,36]. Table 5 summarizes the prevalence data by group and overall averages, showing that 25% of the animals exhibited hepatopancreatic lesions linked to bacterial infections, while 53% displayed WzSV8 inclusion bodies. Notably, BP inclusion bodies were observed in 22% of the samples, a prevalence closely mirroring that of hepatopancreatic damage. The severity of histological damage associated with BP was comparable, with an average lesion grade of 2.3. Lesion severity was assessed on a standardized scale: 0 = no lesions, 1 = lesions, or infection present in <25% of the area, organ, or tissue section, 2 = 25–50%, 3 = 50–75%, and 4 = >75% [12].

PCR analysis revealed a significant discrepancy between histological and molecular findings. While 73% of the samples tested positive for WzSV8 by PCR, only 6% were positive for BP using one of the six primer combinations. This inconsistency was especially pronounced in the larval group analysis, where histology identified BP inclusion bodies in 0.5% of individuals, but PCR failed to detect any positive cases. Given that 1 g of post-larvae—equivalent to at least 200 individuals—was homogenized for PCR, the likelihood of detecting BP should have been much higher, regardless of prevalence. The negative PCR results, despite histological evidence, suggest potential issues with primer sensitivity and specificity, possibly linked to the genetic variability of BP strains. These findings highlight the limitations of current molecular diagnostic techniques and emphasize the need for improved sequencing data to enhance detection accuracy.

These findings suggest a lack of specificity in the primers used for BP detection, likely due to the genetic variability of BP strains across different geographic regions. Primer mismatches at the 3′ end, as reported by Cheng [13], can inhibit amplification, preventing successful PCR detection. The high genetic diversity of BP complicates the development of reliable molecular diagnostics, necessitating improved sequencing data to refine PCR-based detection methods. To address this challenge, the use of nested PCR has been recommended, a strategy successfully employed for Monodon baculovirus (MBV) in penaeid shrimp and other pathogens where enhanced sensitivity has improved detection outcomes.

## 4. Discussion

Baculovirus penaei (BP) is a strictly enteric pathogen that primarily infects the early larval stages of shrimp, including zoea, mysis, and early postlarvae (PL). While BP can also infect later developmental stages, it is most pathogenic to the early stages, where it often causes high mortality. This infection leads to significant reductions in survival rates in nursery and grow-out ponds, with severe implications for shrimp farming. The high mortality associated with BP infection complicates monitoring efforts, as tanks diagnosed as positive are typically discarded, making it difficult to track and manage the spread of the disease effectively.

This has probably contributed to an underestimation of the value of *B. penaei*, at least as an indicator of co-infections [14,15,16,17,18,19,20]. Although in 2009, the WOAH, formerly known as the Office International des Epizooties (OIE), determined that BP no longer met the criteria for inclusion on the list of notifiable diseases, it was officially delisted in May of that year [25]. Due to the incidence within shrimp hatcheries, studies on BP have continued, revealing significant geographic variation among BP strains. This was evidenced through morphometric analysis of BP virion nucleocapsids and strain-specific hybridization probes from the Ecuadorian strain compared to those from North, Central, and South America and Hawaii suggesting the existence of at least three distinct strains of BP [14,18,27,61].

Further genomic studies are crucial to obtaining the complete BP genome, which would enable the development of more specific and sensitive diagnostic tools. The absence of a full genome sequence poses significant limitations in designing robust molecular diagnostics and understanding the genetic diversity of BP across different regions. Primers targeting putative BP cDNA sequences have been developed [26], designing three forward primers (BPA, BPD, and BPE) and three reverse primers (BPF, BPB, and BPG), generating six primer pair combinations capable of amplifying PCR products ranging from 196 to 933 bp. However, Cheng et al. [13] reported the first detection of BP outside the Americas, in northern Taiwan, and highlighted that these primer sets lack specificity. This finding underscores the urgent need for improved sequencing data to refine PCR-based detection methods and address the high genetic diversity among BP strains, which complicates reliable molecular diagnostics. A complete genome sequence would not only enhance detection accuracy but also provide critical insights into BP’s epidemiology, aiding in the development of effective disease management strategies.

Similarly, the PCR results in our study showed a poor correlation with the characteristic polyhedral occlusion bodies of BP observed in histological findings, with only 6% of histologically positive samples testing positive by PCR. In an advanced pathogen monitoring study of *P. vannamei* [35,36], the critical importance of primer selection for achieving reliable PCR outcomes was emphasized. The study attributed inconsistencies in primer performance to the genetic diversity of viruses, which is influenced by factors such as the geographical distribution of hosts, environmental conditions, and viral adaptations to local ecosystems. Collectively, these findings underscore the need for region-specific molecular tools to address challenges in diagnostic precision and accuracy.

Histological analysis consistently revealed hepatopancreatic (HP) lesions associated with bacterial activity, suggesting that these lesions are likely triggered by bacterial exotoxins. These exotoxins may be ingested by shrimp foraging on uneaten feed or organic matter accumulating at the pond or tank bottom. Over time, acute toxic effects from these exotoxins can develop into chronic inflammatory lesions in the HP. Notably, BP was consistently detected as a co-infection alongside bacterial pathogens, raising the possibility that bacterial toxins could create a favorable environment for viral replication and inclusion body formation (Figure 2). This idea finds support in the work by [62], who demonstrated that low-level BP infections in *F. duorarum* were significantly intensified when shrimp were exposed to 1–3 ppb of polychlorinated biphenyls (PCBs). Their findings highlighted how external stressors, such as chemical contaminants (e.g., Aroclor 1254), interact with the host and pathogens to exacerbate disease dynamics. Similarly, bacterial exotoxins may act as stressors that, much like PCBs, promote the progression of viral infections and contribute to the development of HP lesions.

Coinfections of viruses and bacterial pathogens within the hepatopancreas of *P. vannamei* have been documented by Intriago et al. [35,36]. Persistent bacterial infections in the hepatopancreas are a common occurrence in shrimp from Latin America, with pathogens such as WzSV8 and DHPV showing significant prevalence. Furthermore, Intriago identified WzSV8 as a frequent constituent of the viriome in both wild and cultured populations of *P. vannamei* and in wild populations of *P. stylirostris* and *P. monodon* throughout the region.

Coinfections involving BP alongside other pathogens have also been widely observed. For instance, Ref. [21] reported concurrent infections with enteric pathogens (*H. penaei* or NHPB) and systemic pathogens (IHHNV and WSSV) in wild populations of *P. vannamei* and *P. stylirostris* from tidal mangrove ecosystems in Tumbes, Peru. Similarly, Ref. [22] identified BP in post-larvae imported to Central America, noting substantially elevated mortality rates when BP was present alongside NHPB.

These findings, along with the results of the present study, suggest that shrimp in the region likely harbor persistent coinfections as part of their adaptation to environmental and pathogenic stressors [35,36]. A notable relationship between viral and bacterial pathogens in the hepatopancreas highlights the complexity of this phenomenon. This observation aligns with the concept of disease tolerance, which has emerged as an alternative strategy to host resistance in managing viral–bacterial coinfections. Unlike resistance, which seeks to reduce pathogen load, disease tolerance focuses on preserving tissue integrity and mitigating organ damage, thereby enabling survival in the face of infection [63].

However, this tolerance may come at a cost. Chronic infections, once established, could lead to compromised fitness and reduced growth rates. Additionally, there is evidence that these chronic infections, including both bacterial and viral pathogens, can be vertically or horizontally transmitted to offspring. This vertical transmission ensures the persistence of pathogens across generations, potentially creating a continuous cycle of infection within shrimp populations and exacerbating the challenges of managing disease outbreaks in aquaculture.

The results underscore the significant impact of *Baculovirus penaei* (BP) on shrimp health and aquaculture management, particularly when it co-occurs with bacterial and other viral pathogens. The high genetic diversity among BP strains, combined with limited genomic data and inadequate primer specificity, underscores the necessity for region-specific molecular diagnostics. Furthermore, the role of bacterial exotoxins as stressors that facilitate BP replication and lesion formation highlights the intricate dynamics of host–pathogen interactions in shrimp.

This complexity necessitates a comprehensive approach, integrating advanced sequencing, tailored diagnostic tools, and strategies to manage co-infections, thereby promoting sustainable shrimp farming practices. Historically, the presence of BP has received little attention; however, our findings reveal that its occurrence is more consistent than previously assumed. Notably, BP’s presence could serve as a bioindicator, with the observation of tetrahedral occlusion bodies potentially reflecting underlying enteric issues, thus emphasizing its broader significance in aquaculture health management.

## Figures and Tables

**Table 1:** List of pathogens screened in farmed, PL, and broodstock shrimp.

Pathogens	References
Hepanhamaparvovirus (DHPV)	[37]
*Macrobrachium* Bidnavirus (MrBdv)	[38]
Decapod Iridescent Virus 1 (DIV1)	[39]
White Spot Syndrome Virus (WSSV)	[40]
Infectious Hypodermal and Hematopoietic Necrosis Virus (IHHNV)	[41,42,43]
Wenzhou shrimp virus 8 (WzSV8)	[44]
Baculovirus Penaei (BP)	[26,28]
*P. vannamei* nodavirus (PvNV)	[45]
Covert Mortality Nodavirus (CMNV)	[46]
Infectious Myonecrosis Virus (IMNV)	[47]
Yellow Head Virus (YHV)	[48]
Taura Syndrome Virus (TSV)	[49,50]
*Macrobrachium* Nodavirus (MrNV)	[51]
*Spiroplasma*	[52]
*Vibrio* spp. (*Vibrio* specific 16S rRNA gene fragment)	[53]
*Rickettsia*-Like Bacteria (RLB)	[54]
Necrotizing Hepatopancreatitis Bacteria (NHP-B)	[55]
*Ecytonucleospora* [*Enterocytozoon*] *hepatopenaei* (EHP)	[56]
Non-EHP Microsporidia	[57]
Acute Hepatopancreatic Necrosis Disease (AHPND)	[58]

**Table 2:** Primers used in this study for the detection of BP.

Primer	Product	Sequence (5′-3′)	Ta	References
WSSV				
146F1	1447 bp	ACTACTAACTTCAGCCTATCTAG	55 °C	[40]
146R1.		TAATGCGGGTGTAATGTTCTTACGA		
146F2 Nested	941 bp	GTAACTGCCCCTTCCATCTCCA.	55 °C	
146R2 Nested		TACGGCAGCTGCTGCACCTTGT		
DHPV				
H441F1	441 bp	GCATTACAAGAGCCAAGCAG	60 °C	[37]
H441R1		ACACTCAGCCTCTACCTTGT		
HPVnF	265 bp	ATAGAACGCATAGAAAACGCT	55 °C	
HPVnR1		CAGCGATTCATTCCAGCGCCACC		
IHHNV				
389F	389 bp	CGGAACACAACCCGACTTTA	55 °C	[41]
389R		GGCCAAGACCAAAATACGAA		
77012F	356 bp	ATCGGTGCACTACTCGGA	55 °C	[42]
77353R		TCGTACTGGCTGTTCATC		
392F	392 bp	GGGCGAACCAGAATCACTTA	55 °C	[43]
392R		ATCCGGAGGAATCTGATGTG		
309F	309 bp	TCCAACACTTAGTCAAAACCAA	55 °C	[41]
309R		TGTCTGCTACGATGATTATCCA		
MrBidnavirus				
MrBdv-L	392 bp	GCATTAATGGATTGGGAAGG	53 °C	[38]
MrBdv-R		TCGATGTCTGGATGACCGTA		
DIV1				
SHIV-F1	457 bp	GGGCGGGAGATGGTGTTAGAT	59 °C	[39]
SHIV-R1		TCGTTTCGGTACGAAGATGTA		
SHIV-F2	129 bp	CGGGAAACGATTCGTATTGGG	59 °C	
SHIV-R2		TTGCTTGATCGGCATCCTTGA		
PvNV				
PvNV339F	339 bp	CTGTCTCACAGGCTGGTTCA	55 °C	[45]
PvNV339R		CCGTTTGAATTTCAGCAACA		
PvNV246NF	246 bp	CAAAACTGTGCCTTTGATCG	60 °C	
PvNV246NR		GCCTTATCCACACGAACGTC		
IMNV				
4587F	328 bp	CGACGCTGCTAACCATACAA	60 °C	[47]
4914R		ACTCGGCTGTTCGATCAAGT		
4725NF	139 bp	GGCACATGCTCAGAGACA	65 °C	
4863NR		AGCGCTGAGTCCAGTCTTG		
CMNV				
CMNV-7F1	619 bp	AAATACGGCGATGACG	45 °C	[46]
CMNV-7R1		ACGAAGTGCCCACAGAC		
CMNV-7F2	165 bp	CACAACCGAGTCAAACC	50 °C	
CMNV-7R2		GCGTAAACAGCGAAGG		
TSV				
9992 F	231 bp	AAGTAGACAGCCGCGCTT	60 °C	[49,50]
9195 R		TCAATGAGAGCTTGGTCC		
7171 F	341 bp	CGACAGTTGGACATCTAGTG	60 °C	
7511 R		GAGCTTCAGACTGCAACTTC		
YHV				
YHV GY1	794 bp	GACATCACTCCAGACAACATCTG	66 °C	[48]
YHV GY4		GTGAAGTCCATGTGTGTGAGACG		
YHV GY2	406–277 bp	CATCTGTCCAGAAGGCGTCTATGA		
YHV Y3		ACGCTCTGTGACAAGCATGAAGTT	66 °C	
YHV G6		GTAGTAGAGACGAGTGACACCTAT		
YHV GY5		GAGCTGGAATTCAGTGAGAGAACA		
WzSV 8				
428 F BIOT		ATGCCTCTGGAAAGCGATAC	60 °C	[44]
428 R BIOT	482 bp	GGTGTTAGATCGCTCCTTCTTC		
168 F BIOT Nested		GAAAGCGATACTCCTACGACAG	60 °C	
168 R BIOT Neste	168 bp	TCTTGAGTTTGAGGAAGGTGAG		
MrNV				
fragment1F	1486 bp	GTTAAACGTTTTGTTTTCTAGC	50 °C	[51]
fragment1R		ACACCTACATTCGCTTCGGG		
fragment2-F	1736 bp	CCCGAAGCGAATGTAGGTGT	50 °C	
fragment2-R		CGAAAGAGTGAAGGAGACTTGG		
RNA2-fragm1F	664 bp	CCCATCATGTGCTAGATATGAC	50 °C	[51]
RNA2-fragm1R		AGGCAGGCTACGTCACAAGT		
RNA2-fragm2-F	534 bp	ACTTGTGACGTAGCCTGCCT	50 °C	
RNA2-fragm2-R		AAAGGATATTCGATATTCTATC		
EHP				
SWP_1F		TTGCAGAGTGTTGTTAAGGGTTT		
SWP_1R	514 bp	CACGATGTGTCTTTGCAATTTTC	58 °C	
SWP_2F Nested		TTGGCGGCACAATTCTCAAACA		[56]
SWP_2R Nested	148 bp	GCTGTTTGTCTCCAACTGTATTTGA	64 °C	
Non-EHP Microsporidia				
TS1	600 bp	GTCGGAATTCGCCAGCAGCCGCGGT	55 °C	[57]
TS2		CAGCGGATCCGTCAAATTAAGCCGC		
Rickettsia				
BACT F	1500 bp	CCGAATTCGTCGACAACAGAGTTTGATCCTGGCTCAG	45 °C	[54]
BACT R		CCCGGGATCCAAGCTTACGGCTACCTTGTTACGACTT		
NHPB				
NHPF2	379 bp	CGTTGGAGGTTCGTCCTTCAGT	72 °C	[55]
NHPR2		GCCATGAGGACCTGACATCATC		
Spiroplasma				
CSF:5′	269 bp	TAGCCGAACTGAGAGGTTGA	60 °C	[52]
CSR:5′		GATAACGCTTGCCACCTATG		
Vibrio	120 bp			
Vib-F		GGCGTAAAGCGCATGCAGGT	55 °C	[53]
Vib2-R		GAAATTCTACCCCCCTCTACAG		
**AHPND**	1269 bp			
AP4F1		ATGAGTAACAATATAAAACATGAAAC	55 °C	[58]
AP4R1	230–357 bp	ACGATTTCGACGTTCCCCAA		
AP4F2 Nested	1142–1269 bp	TTGAGAATACGGGACGTGGG	55 °C	
AP4R2 Nested		GTTAGTCATGTGAGCACCTTC		
**BP**				
BPA	560 bp	GATCTGCAAGAGGACAAACC	61 °C	[26]
BPB		ATCGCTAAGCTCTGGCATCC		
BPA	196 bp	GATCTGCAAGAGGACAAACC	61 °C	
BPF		TACCCTGCATTCCTTGTCGC		
BPA	933 bp	GATCTGCAAGAGGACAAACC	61 °C	
BPG		ATCCTGTTTCCAAGCTCTGC		
BPD	207 bp	TGTTCTCAGCCAATACATCG	61 °C	
BPB		ATCGCTAAGCTCTGGCATCC		
BPD	580 bp	TGTTCTCAGCCAATACATCG	61 °C	
BPG		ATCCTGTTTCCAAGCTCTGC		
BPE	221 bp	TACATCTTGGATGCCTCTGC	61 °C	
BPG		ATCCTGTTTCCAAGCTCTGC		
6581	644 bp	TGTAGCAGCAGAGAAGAG	61 °C	[28]
6582		CACTAAGCCTATCTCCAG		

**Table 3:** Different organ samples used for DNA extraction.

Pathogens	Target Tissues
IHHNV	2 pleopods per animal pool of 5 animals.
PvNV	2 pleopods per animal pool of 5 animals.
Spiroplasma	DNA pool of 2 pleopods per animal, pool of 5 animals; 10 gill pools of animals; whole hepatopancreas pools of 5 animals
*Vibrio* spp.	DNA pool of 2 pleopods per animal pool of 5 animals. 0.5 g of tail muscle per animal pool 5 of animals
WSSV, TSV, MrNV, IMNV, YHV	10 gills per animal pool of 5 animals
DHPV, DIV1, WzSV8, RLB, NHPB, EHP, AHPND, BP	Whole hepatopancreas pool of 5 animals
Non EHP microsporidia, CMNV	0.5 g of tail muscle per animal pool of 5 animals
Any pathogen in post larvae	1 g of larvae regardless of the stage.

**Table 4:** Summary of the positive PCR results. A comprehensive list of all analyses performed is provided in the Materials and Methods Section and in the table’s footnote.

**Sample**	**Location**	**Date**	**DNA Virus**								
			**DHPV ^1^**		**WSSV ^2^**	**IHHNV ^3^**					
						**309 F/R (309 bp)**	**392 F/R (392 bp)**	**389 F/R (389 bp)**	**77012F/77353R (356 bp)**	**EVE ^4^**	**Virus ^5^**
1	Farm	07/17/2023	**−**		**+**	**−**	**−**	**−**	**−**	**−**	**-**
2	Farm	07/17/2023	**−**		**−**	**−**	**−**	**−**	**−**	**−**	**-**
3	Farm	07/17/2023	**−**		**+**	**−**	**−**	**−**	**−**	**−**	**-**
4	Farm	07/17/2023	**−**		**+**	**−**	**−**	**−**	**−**	**−**	**-**
5	Farm	07/17/2023	**−**		**+**	**−**	**−**	**−**	**−**	**−**	**-**
6	Farm	07/17/2023	**−**		**+**	**−**	**−**	**−**	**−**	**−**	**-**
7	Farm	07/17/2023	**−**		**+**	**−**	**−**	**−**	**−**	**−**	**-**
8	Farm	07/17/2023	**−**		**+**	**−**	**−**	**−**	**−**	**−**	**-**
9	Farm	07/17/2023	**−**		**+**	**−**	**−**	**−**	**−**	**−**	**-**
10	Farm	07/17/2023	**−**		**+**	**−**	**−**	**−**	**−**	**−**	**-**
11	Farm	07/17/2023	**−**		**+**	**−**	**−**	**+**	**−**	**−**	**-**
12	Farm	07/17/2023	**−**		**−**	**−**	**−**	**−**	**−**	**−**	**-**
13	Broodstock	1/10/2024	**+**		**−**	**−**	**−**	**−**	**−**	**−**	**-**
14	Broodstock	1/10/2024	**−**		**−**	**−**	**−**	**−**	**−**	**−**	**-**
15	Broodstock	1/10/2024	**+**		**−**	**−**	**−**	**−**	**−**	**−**	**-**
16	Broodstock	1/10/2024	**+**		**−**	**−**	**−**	**−**	**−**	**−**	**-**
17	Broodstock	1/10/2024	**+**		**−**	**−**	**−**	**−**	**−**	**−**	**-**
18	Broodstock	1/10/2024	**+**		**−**	**−**	**−**	**+**	**−**	**+**	**-**
19	Broodstock	1/10/2024	**+**		**−**	**−**	**−**	**−**	**−**	**−**	**-**
20	Broodstock	1/10/2024	**+**		**−**	**−**	**−**	**−**	**−**	**−**	**-**
21	Broodstock	1/10/2024	**+**		**−**	**−**	**−**	**−**	**−**	**−**	**-**
22	Broodstock	1/10/2024	**−**		**−**	**+**	**+**	**+**	**−**	**+**	**-**
23	Broodstock	1/10/2024	**−**		**−**	**+**	**+**	**+**	**+**	**−**	**+**
24	Broodstock	1/10/2024	**−**		**−**	**+**	**+**	**+**	**+**	**−**	**+**
25	Broodstock	1/10/2024	**+**		**−**	**+**	**+**	**+**	**−**	**+**	**-**
26	Broodstock	1/10/2024	**−**		**−**	**−**	**−**	**−**	**−**	**−**	**-**
27	Broodstock	1/10/2024	**+**		**−**	**−**	**−**	**−**	**+**	**+**	**-**
28	Broodstock	1/10/2024	**−**		**−**	**+**	**−**	**+**	**+**	**+**	**-**
29	PL	1/10/2024	**−**		**−**	**−**	**−**	**−**	**−**	**−**	**-**
30	PL	1/10/2024	**−**		**−**	**−**	**−**	**−**	**−**	**−**	**-**
31	PL	1/10/2024	**−**		**−**	**−**	**−**	**−**	**−**	**−**	**-**
32	PL	1/10/2024	**−**		**−**	**−**	**−**	**−**	**−**	**−**	**-**
33	PL	1/10/2024	**−**		**−**	**−**	**−**	**−**	**−**	**−**	**-**
% of prevalence			**30%**		**30%**	**15%**	**12%**	**18%**	**12%**	**15%**	**6%**
**Sample**	**Location**	**Date**	**DNA Virus**							**RNA Virus**	
			**BP ^6^**							**WzSV8 ^7^**	***Vibrio* spp. ^8^**
			**BPA/BPF (196 bp)**	**BPA/BPB (560 bp)**	**BPA/BPG (933 bp)**	**BPD/BPB (207 bp)**	**BPD/BPG (580 bp)**	**BPE/BPG (221 bp)**	**6581/6582 (644 bp)**		
1	Farm	07/17/2023	**−**	**−**	**−**	**−**	**−**	**−**	**−**	**-**	**-**
2	Farm	07/17/2023	**−**	**−**	**−**	**−**	**−**	**−**	**−**	**-**	**-**
3	Farm	07/17/2023	**−**	**−**	**−**	**−**	**−**	**−**	**−**	**+**	**-**
4	Farm	07/17/2023	**−**	**−**	**−**	**−**	**−**	**−**	**−**	**-**	**-**
5	Farm	07/17/2023	**−**	**−**	**−**	**−**	**−**	**−**	**−**	**+**	**+**
6	Farm	07/17/2023	**−**	**−**	**−**	**−**	**−**	**−**	**−**	**-**	**+**
7	Farm	07/17/2023	**−**	**−**	**−**	**−**	**−**	**−**	**−**	**-**	**-**
8	Farm	07/17/2023	**−**	**−**	**−**	**−**	**−**	**−**	**−**	**-**	**-**
9	Farm	07/17/2023	**−**	**−**	**−**	**−**	**−**	**−**	**−**	**-**	**-**
10	Farm	07/17/2023	**+**	**−**	**−**	**−**	**−**	**−**	**−**	**+**	**-**
11	Farm	07/17/2023	**−**	**−**	**−**	**−**	**−**	**−**	**−**	**-**	**-**
12	Farm	07/17/2023	**−**	**−**	**−**	**−**	**−**	**−**	**−**	**+**	**-**
13	Broodstock	1/10/2024	**−**	**−**	**−**	**−**	**−**	**−**	**−**	**+**	**-**
14	Broodstock	1/10/2024	**−**	**−**	**−**	**−**	**−**	**−**	**−**	**+**	**-**
15	Broodstock	1/10/2024	**−**	**−**	**−**	**−**	**−**	**−**	**−**	**+**	**-**
16	Broodstock	1/10/2024	**−**	**−**	**−**	**−**	**−**	**−**	**−**	**+**	**-**
17	Broodstock	1/10/2024	**−**	**−**	**−**	**−**	**−**	**−**	**−**	**+**	**-**
18	Broodstock	1/10/2024	**−**	**−**	**−**	**−**	**−**	**−**	**−**	**+**	**-**
19	Broodstock	1/10/2024	**−**	**−**	**−**	**−**	**−**	**−**	**−**	**+**	**-**
20	Broodstock	1/10/2024	**−**	**−**	**−**	**−**	**−**	**−**	**−**	**+**	**+**
21	Broodstock	1/10/2024	**−**	**−**	**−**	**−**	**−**	**−**	**−**	**+**	**+**
22	Broodstock	1/10/2024	**−**	**−**	**−**	**−**	**−**	**−**	**−**	**+**	**+**
23	Broodstock	1/10/2024	**−**	**−**	**−**	**−**	**−**	**−**	**−**	**+**	**-**
24	Broodstock	1/10/2024	**+**	**+**	**−**	**−**	**−**	**−**	**−**	**+**	**-**
25	Broodstock	1/10/2024	**−**	**−**	**−**	**−**	**−**	**−**	**−**	**+**	**+**
26	Broodstock	1/10/2024	**−**	**−**	**−**	**−**	**−**	**−**	**−**	**-**	**-**
27	Broodstock	1/10/2024	**−**	**−**	**−**	**−**	**−**	**−**	**−**	**+**	**-**
28	Broodstock	1/10/2024	**−**	**−**	**−**	**−**	**−**	**−**	**−**	**+**	**-**
29	PL	1/10/2024	**−**	**−**	**−**	**−**	**−**	**−**	**−**	**+**	**-**
30	PL	1/10/2024	**−**	**−**	**−**	**−**	**−**	**−**	**−**	**+**	**-**
31	PL	1/10/2024	**−**	**−**	**−**	**−**	**−**	**−**	**−**	**+**	**-**
32	PL	1/10/2024	**−**	**−**	**−**	**−**	**−**	**−**	**−**	**+**	**-**
33	PL	1/10/2024	**−**	**−**	**−**	**−**	**−**	**−**	**−**	**+**	**-**
**% of prevalence**			**6%**	**3%**						**73%**	**18%**

^1^ [37]; ^2^ [40]; ^3^ [41,42,43]; ^4^ IHHNV as EVE (endogenous viral element); ^5^ IHHNV as virus; ^6^ [26,28]; ^7^ [44]; ^8^ [53].

**Table 5:** Histological average of lesions of affected animals in farms and hatchery.

	**Ave ^4^ (g)**	**n ^5^**	**Collapsed**		**Hemocytic**		**Muscle**		**Exoskeleton**	
			**Hepatopancreas ^1^**		**Enteritis**		**Necrosis**		**Melanization**	
			**% ^6^**	**Grade ^7^**	**% ^6^**	**Grade ^7^**	**% ^6^**	**Grade ^7^**	**% ^6^**	**Grade ^7^**
Broodstock	50.60	10	58.0	1.8	-		90.0	2.5	100.0	3.0
Farm	40.75	12	13.4	1.5	16.5	1.00	39.2	1.5	33.3	1.5
PL	0.004	500	2.3	3.8	-		-		-	
Average			24.6	2.4	5.5	1.0	43.1	2.0	44.4	2.3
			21.80	2.20	8.30	1.00	42.10	1.80	41.70	2.30
	**Alteration in**		**DHPV ^2^**		**WzSV8 ^2^**		**BP ^2^**		**Greg+Nemat ^3^**	
	**Lymphoid Organ**									
	**% ^6^**	**Grade ^7^**	**% ^6^**	**Grade ^7^**	**% ^6^**	**Grade ^7^**	**% ^6^**	**Grade ^7^**	**% ^6^**	**Grade ^7^**
Broodstock	10.0	1.0	-		70.0	1.5	50.0	3.5	-	
Farm	58.3	2.0	33.3	3.5	75.0	2.3	16.7	2.5	16.5	1.0
PL	-		-		12.6	1.0	0.5	1.0	-	
Average	22.8	1.5	11.1	3.5	52.5	1.6	22.4	2.3	5.5	1.0

^1^ Average hepatopancreas abnormalities (cell sloughing, hemocytic–melanized and necrotic tubules, atrophied/destroyed tubules, hemocytic enteritis); ^2^ viral inclusion bodies or VIB. ^3^ Sum of gregarines and nematodes. ^4^ Average weight (g). ^5^ Total number of animals analyzed. ^6^ Percentage of prevalence. ^7^ The average of a grading system of severity was adopted from [12] and simplified as follows: 0 = no lesions, 1 = lesions or infection present in <25% of area or organ or tissue section, 2 = lesions or infection present in 25–50% of area or organ or tissue section, 3 = lesions or infection present in 50–75% of area or organ or tissue section, 4 = lesions or infection present in >75% of area or organ or tissue section [12]. Superscript numbers refer to the table.

## Data Availability

The datasets generated and analyzed during the current study are available from the corresponding author upon reasonable request. Due to the sensitive nature of location-specific data, access may be restricted to ensure compliance with ethical, privacy, or commercial considerations.

## Abbreviations

*Penaeus vannamei*—Pv.; Baculovirus penaei—single enveloped nucleopolyhedron virus (BP—PvSNPV); Hepatopancreas—HP; Wenzhou shrimp virus 8—WzSV8; Hepanhamaparvovirus—DHPV; Postlarvae—PL; Viral inclusion—VIN; World Organization for Animal Health—WOAH.
